# Proposal of a New Orange Selection Process Using Sensory Panels and AHP

**DOI:** 10.3390/ijerph18073333

**Published:** 2021-03-24

**Authors:** Amparo Baviera-Puig, Mónica García-Melón, María Dolores Ortolá, Isabel López-Cortés

**Affiliations:** 1 Department of Economics and Social Sciences, Universitat Politècnica de València, Camino de Vera s/n, 46022 Valencia, Spain; 2 INGENIO (CSIC-UPV), Universitat Politècnica de València, Camino de Vera s/n, 46022 Valencia, Spain; mgarciam@dpi.upv.es; 3 Institute of Food Engineering for Development, Universitat Politècnica de València, Camino de Vera s/n, 46022 Valencia, Spain; mdortola@tal.upv.es; 4 COMAV, Department of Plant Production, Universitat Politècnica de València, Camino de Vera s/n, 46022 Valencia, Spain; islocor@upv.es

**Keywords:** retailing, supplier selection process, consumer behavior, healthy diet, multicriteria decision making, food waste, aroma, juiciness, sweetness, acidity

## Abstract

Although the consumption of fruits and vegetables is being promoted by different institutions as a key question of public health, their consumption is decreasing and their waste is increasing. To address this situation, we propose to include the consumer’s perception of the quality (from a sensory point of view) of a fruit, in particular Valencian oranges, in the supplier’s selection process by retailers. To do so, we use a combination of consumer and trained sensory panels and Analytic Hierarchy Process (AHP). This approach is completely novel in the literature. According to the expert panel, the most important criteria when evaluating the quality of an orange are fruity smell, juiciness, sweetness and acidity. These criteria are related to the freshness and taste of the oranges. Consumers found the methodology proposed useful and easy to develop. The application of the AHP methodology has helped to facilitate a participatory discussion among consumers on the concept of the quality of the oranges. The methodology proposed can help the agrifood sector in different ways up and down the supply chain. Specially, it can contribute to better meet consumer’s demands, increasing the consumption of fruits and vegetables and reducing its waste.

## 1. Introduction

Fruits and vegetables are essential components of a healthy diet, and sufficient daily consumption could contribute to the prevention of major diseases such as cardiovascular diseases and some cancers [1,2]. By May 2004, the 57th World Health Assembly adopted the World Health Organization’s Global Strategy on Diet, Physical Activity and Health (DPAS), which emphasizes increased consumption of fruits and vegetables as one of the recommendations to be taken into account when developing national dietary policies and guidelines for both the population and individuals [3].

For this reason, in recent years the Public Administration has launched different campaigns to encourage the consumption of fruit and vegetables, both at European and national level. In Spain, there have been different campaigns promoted by the Ministry of Agriculture, Fisheries and Food [4]. The most recent, presented in the European Parliament on 11 September 2019, is the promotional campaign “CUTE, Cultivating the Taste of Europe”, promoted by the European Fruit and Vegetable Association (EUCOFEL) and relevant fruit and vegetable associations in Spain, France, Greece and Poland, with the aim of disseminating the excellent qualities of European fruits and vegetables, grown in greenhouses and in the open air, raising awareness of their well-being for consumers [5].

At world level, this is The International Year of Fruits and Vegetables 2021 (IYFV) declared by the United Nations General Assembly [6]. The objectives are, on one side, to highlight the benefits of fruits and vegetables consumption and its contribution to balanced and healthy diets and lifestyles and, on the other side, to increase the sustainability of its production and trade and to reduce the loss and waste of fruits and vegetables. Additionally important are the actions carried out by other organizations, such as five a day, an international organization that brings together organizations in more than 40 countries on five continents, which carry out activities to promote the consumption of five portions of fresh fruit and vegetables per day [7,8,9].

However, despite all these efforts, fresh fruit consumption in households is gradually decreasing. According to the latest report published by our Ministry of Agriculture, Fisheries and Food on Food Consumption in Spain 2019 [10], in 2019 this market lost 11.1% of the volume of purchases compared to 2013. As an example, the consumption of oranges (the most consumed fresh fruit in Spain according to this report) fell by 4.9% with respect to 2018 consumption, and in terms of turnover, the fall was much greater, reaching a value of 8.9% because of the fall in the average price (5.3%). Furthermore, in households, the waste of unprocessed products has been increasing compared to processed products, with fresh products such as fruit and vegetables leading the ranking (44.3% of the volume of waste). In distribution companies, 27% of waste is attributed to variations in sales and failures in planning [11]. It is paradoxical that, on the one hand, a healthy diet based on the consumption of fruit and vegetables is being promoted and that, on the other hand, the waste of this type of products is increasing.

This current situation does not stem from a technical agronomic problem but from a socio-economic problem linked to the difficulty of satisfying the markets, i.e., how the supply of fruit can best meet the existing demand. As a matter of fact, consumers are willing to consume healthier products without compromising on taste [12]. In addition, consumers play a crucial role in food waste via their own household and in-store choices [13,14]. Their actual or anticipated food perceptions and food purchase behaviors influence stakeholder decision making along the whole supply chain. So, the interaction between the retailer and the consumer determines food waste both up and down the supply chain [15,16].

The question therefore arises as to whether, despite the campaigns and the well-known innumerable benefits of fresh fruit consumption, we are offering the consumer what he or she really expects. To answer this question, we have to take into account several actors. First, there is the retailer who delivers the fresh fruit to the consumer via different suppliers. Second, there is the consumer himself who selects the fresh fruit according to his/her preferences. Although it is obvious that the large-scale retailer looks for quality when choosing the fresh fruit [17], we must be able to understand how the consumer perceives this quality in order to be able to offer it to them. Although there is a large literature on the selection of suppliers by retailers in this product [18,19,20], the gap that we aim to address in this article is to include the consumer’s perception of the product quality in the supplier selection by the retailer. If retailers are able to choose suppliers that offer the product that consumers are looking for, the benefit is twofold: (i) consumption of fruit and vegetables can increase as it is adapted to consumers’ demands; (ii) the waste of this type of product may be reduced.

The aim of the paper is to create a consumer supplier selection model for fresh fruit, in particular Valencian oranges, for the retailer. This general objective is divided into the following specific objectives: (i) to analyze the consumer’s decision process when choosing a fruit, specifically oranges; (ii) to determine the importance of each of the criteria considered during this process in order to evaluate the quality of the orange; and (iii) to develop the fresh fruit supplier selection model based on the above information.

As a methodology, we have used a combination of two different methods. First, we used consumer and trained sensory panels [21] to select the variables necessary for the orange tasting and, second, we used the Analytic Hierarchy Process (AHP), a multicriteria decision making method, to elaborate a prioritization model of orange quality based on the selected variables [22,23]. In this way, once the oranges have been prioritized, we will also obtain the prioritization of their suppliers. In the food supply chain literature, it is very common to find multicriteria models such as AHP [24], ANP [25], TOPSIS [26], DEMATEL [27] or PROMETHEE [28]. However, we have not found articles that combine sensory analysis with multicriteria methods. Therefore, the approach of this article is novel as it proposes to include consumer perception of quality using trained and consumer sensory panels in the multicriteria process of selection of oranges and their suppliers.

The article is structured as follows. First, the theoretical framework and the research questions are presented. Second, the methodology used (sensory panels and AHP) is explained. Next, the results and their relationship with the previous literature are discussed and, finally, the conclusions.

## 2. Theoretical Framework

To achieve the objectives, we need to understand the consumer’s evaluation process of a fruit. Consumers believe both food safety and quality are important to food in general, but they pay relatively more attention to food quality when purchasing a product [29]. In order to make a choice, the different alternatives available to the consumer must first be evaluated. This assessment consists of processing information from different sources on factors affecting the buyer’s perception of quality [29,30]. According to [31], the perceived quality could be defined as the consumer’s judgment about a product’s overall excellence or superiority. Grunert et al. [32] identified two key moments for quality evaluation: (i) before purchase, when consumers form quality expectations; (ii) and after purchase, when consumers undergo the quality experience. At the time of purchase, consumers choose those products that they think will best meet their preferences based on the information available. When purchasing fresh fruit, the information consumers have is derived from inspection of perceptions of extrinsic and intrinsic attributes and their own experience of the fruit after purchase on previous occasions [33]. Petrescu et al. [34] found that consumers use freshness, taste and appearance to assess food quality. In the case of the Valencian oranges, the following research question is proposed:

RQ1: What are the most relevant criteria for a consumer when choosing oranges?

As not all consumers are the same [35], there may be differences in the evaluation of oranges. The perception of the quality of the same fruit may be different depending on the profile of the person [30]. The second research question tries to address this diversity:

RQ2: Are the consumers’ judgements homogenous?

Retailers should therefore consider all these factors when choosing fresh fruit suppliers. However, in the literature, there are different proposals in the food industry that include green criteria [18,20], price, food safety concerns [19] and logistics [36]. Although all of them take food quality into account [17], none of them include it from a sensory point of view. Sensory quality relates both intrinsic product attributes and the interaction between the product and the consumer [37]. So, the consumer should play a role in the assortment choices by the retailers, as well as for their choices of suppliers. This is why we suggest including the proposed methodology (consumer and trained sensory panels and AHP) to refine the process of supplier selection by retailers and better adapt to consumer’s demands. For this reason, the last research question is suggested:

RQ3: Is the orange selection process suitable for the retailers in their supplier selection?

## 3. Materials and Methods

### 3.1. Sensory Panels

In this research, a trained sensory panel and a consumer sensory panel were carried out. Each session required a minimum of eight tasters for the data to have statistical value and significance. The tastings took place in a suitable room that, in addition to being odor-free, was sufficiently isolated and comfortable, and it was under a controlled room temperature. Inside the room, there were different workstations, called booths, with front and side walls that isolated each taster from the others. There was also a place where samples could be prepared and material cleaned.

The trained panel was chosen after passing different training tests, which were carried out in several sessions. During these training sessions we performed the recognitions and qualification of the visual and tactile attributes that are characteristic of the fruit, both positive and negative [38]. In the training sessions, olfactory and taste tests were carried out, with artificial aromas prepared, known as “nose”. They were always aromas related to the fruit to be tasted, and taste tests that prepared different acidity and sweetness scales, among others. The tastings were carried out with a tasting sheet that defined the attributes, which used the typology of adjectives of sensory analysis of fresh fruits [39]. In the sharing that took place at the end of each tasting session, the tasters presented their scores, opinions and points of view, without modifying in any case, the data that were noted on the tasting sheet. Consensus was obtained from the report of each of the samples. Fourteen expert tasters participated in the research; they were 8 women and 6 men. They belonged to the following areas: marketing, production, research and consumer. Some of them were trained tasters in other products. With this profile distribution, we can assess all the important aspects of a fresh fruit.

In contrast, in the consumer sensory panel, consumers were randomly selected to carry out this research. They were all convened at the same time, specifying that they could not eat, drink or smoke one hour before the tasting session. Once in the tasting room, the procedure to be followed in the evaluation of the fresh oranges was explained to them. The orange samples were already prepared at each work station before the tasting session began and the tasters entered. Four samples of oranges were analyzed, and their arrangement in each booth was the same for all tasters. The temperature of the samples was appropriate. All tasters started at the same time and in the order established by the panel leader. Throughout the tasting, they had to fill in the tasting sheet. During the tasting session, they were asked not to talk to anyone as they may distract and influence the judgement of other tasters. They were also asked to notify the panel leader if they had any problems with the samples or any questions about the assessment procedure. In the research, 23 consumers participated.

### 3.2. Organoleptic Session Assessment Sheet

In both panels (trained and consumers), the tasting session was carried out using the tasting sheet, which allowed establishing the sensations transmitted by the oranges during the sensory analysis. The established phases were tactile, visual, olfactory and gustatory. In the tactile phase, it was in our interest to determine how firm the skin of the oranges was, so the tasters assessed the roughness of the samples to the touch [40] and also evaluated the presence of any touch defects.

In the visual phase, the color of both the skin and the pulp of the samples was studied. To this effect, there was a color scale in the taste sheet [41] adapted to the colors of the citrus fruits of the Mediterranean climate zone. The possible visual defects were also assessed in this stage. How easy it was to peel the orange belonged to the visual phase, so the ease of peeling the orange was assessed at this time of the organoleptic tasting [42], as well as the compaction of the slices and the presence of seeds, which are very important aspects for the final consumer.

In the gustatory phase it is interesting to study the sugar found in the samples [39,43] by making a quantitative assessment of the intensity of the sweetness. We also took into account the level of acidity that the samples presented, in a similar way to the article by [43]. The balance in an orange depends on the aforementioned sweetness and acidity, but the bitter and astringent taste must be valued, as it was studied in the article by [39], in order to have a joint understanding of the parameters.

Next, we achieved a global vision of the gustatory phase with the evaluation of negative attributes. In this sense, the presence of notes of fermentation and mold were taken into account [43], rating its intensity. In this phase, we were also interested in the duration of the taste of the sample after tasting, that is, the permanence of the residue in the mouth. This attribute was also evaluated in the article by [39]. Finally, the juiciness of the orange in the mouth was evaluated, trying to analyze how juicy the orange was, regardless of its taste. For all this evaluation, both in oranges and in juice, positive and negative attributes were rated; this is why a numerical scale was used in a similar way to the one used in the article by [44].

In the olfactory phase, it is interesting to study the presence of terpenes [45], so the tasters valued the intensity of herbaceous and fruity aromas. These positive attributes along with the negative olfactory attributes, such as fermented aromas, were quantified numerically in a similar way to the one in the article by [39]. In addition to the numerical quantitative evaluation in the olfactory and gustatory phases, tasters could complement their evaluation in a descriptive way [46], writing the observations they considered appropriate at the end.

### 3.3. Analytic Hierarchy Process

As mentioned in previous sections, the sensory quality of a fruit depends on many variables that are grouped into four major qualities: appearance, texture, smell and taste. It is accordingly a problem of a multicriteria nature whose variables (criteria) can be hierarchically structured. Therefore, in order to present consumers with an alternative selection method to the tasting session that does not involve tasting knowledge or previous training, we have proposed the use of the multicriteria decision technique Analytic Hierarchy Process (AHP) [47].

AHP is based on the fact that the inherent complexity of a multiple criteria evaluation problem can be solved through the construction of hierarchic structures consisting of a goal, criteria and alternatives. In each hierarchical level, paired comparisons are made with judgments using numerical values taken from the AHP absolute fundamental scale of 1–9. These comparisons lead to dominance matrices from which ratio scales are derived in the form of principal eigenvectors. These matrices are positive and reciprocal (aij = 1/aji). The synthesis of AHP combines multidimensional scales of measurement into a single one-dimensional scale of priorities. Hence, for each fruit analyzed, a one-dimensional AHP weight will be obtained, which will lead us to the priority of each orange. The AHP method has the additional advantage of being easy to explain to the consumers who have to assess the different oranges in a simple and systematic way. It also provides a very good decision-making framework when working with qualitative variables, such as the organoleptic properties of the fruits.

The method is one of the most extended multicriteria decision making techniques. AHP is being currently applied in the food industry field [48,49,50], and it adapts very well to the hierarchy of criteria proposed by the experts because all of them are of the qualitative type. AHP deals very well with qualitative criteria or uncertainty scenarios [51]. It also has the additional advantage of being easy to explain to the experts and consumers that have to assess the different criteria and oranges in a simple and systematic way. More details on the AHP can be found in [47].

### 3.4. Methodology Proposed

**Figure 1:**
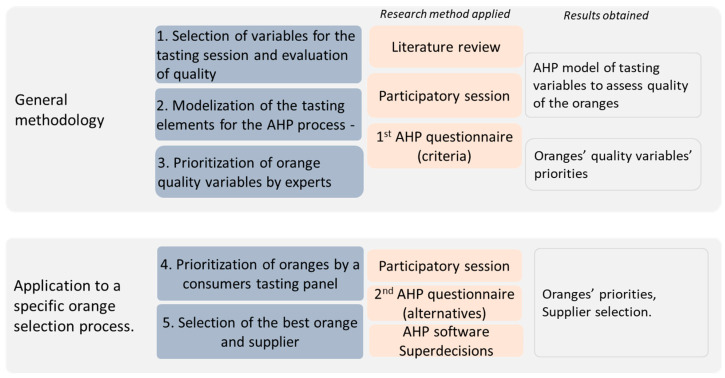
Methodological approach.

The proposed methodology is structured as shown in Figure 1. Following, we detail all the methodology steps adding the results of the case study of the oranges for a better understanding.

## 4. Case Study and Results—Evaluation of Seasonal Oranges

First, we will describe thoroughly the stages of the general methodology, aimed at obtaining a model of prioritization of oranges based on their quality useful in any process of selection of orange suppliers.

### 4.1. Selection of the Variables for the Tasting Session

According to the literature review as well as the work carried out during the participatory session with the expert tasters, as indicated in Section 3.2., the variables or criteria used for the selection process were

- tactile phase (touch): firmness, skin roughness, and defects in touch;
- visual phase (aspect): visual defects, skin color, pulp color, easy peeling, presence of seeds and slices compaction;
- olfactory phase (smell): herbaceous and fruity;
- gustatory phase (flavor): sweetness, acidity, bitter, astringent, residue in mouth, and juiciness.

### 4.2. Modelization of the Orange Quality Evaluation Criteria for the AHP Process

**Figure 2:**
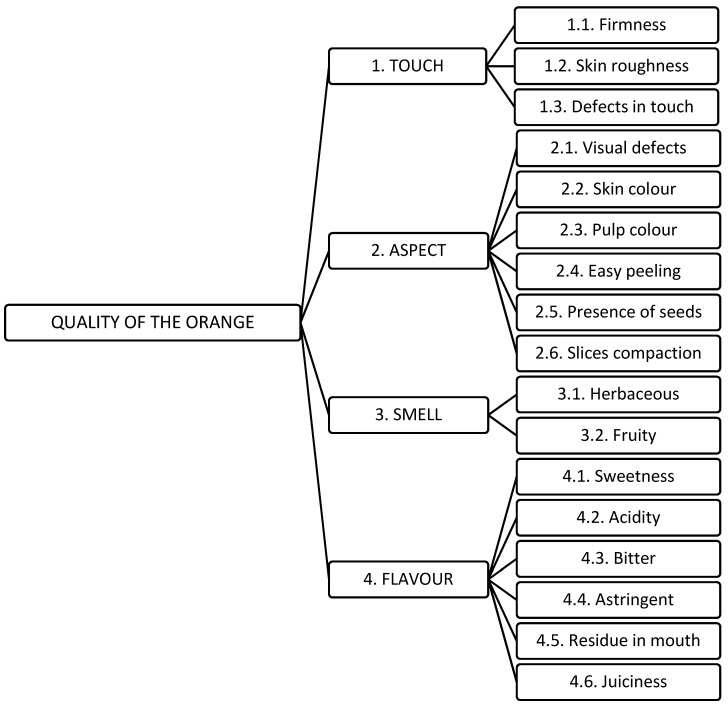
AHP structure of the oranges’ prioritization process.

The criteria were then organized in the form of an AHP hierarchy for their prioritization. The result is presented in Figure 2.

### 4.3. Prioritization of Orange Quality Criteria by Experts

For this stage a participatory session with the tasting experts was arranged. All of them had a wide experience in orange tasting for a Valencian company that distributes food products. Since the criteria that appear in the AHP model are very similar to the ones they usually use in their organoleptic session assessment sheet, it was not necessary to explain to them the meaning of the variables to evaluate. However, it was necessary to introduce them to the concept of AHP and its related paired comparisons, so that they would understand the format of the questionnaire to be handed to them. The process of explaining the questionnaire took about 30 min, and after that all the experts were able to complete it. This process took approximately another 30 min.

**Figure 3:**
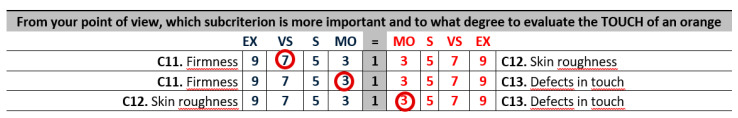
Example of a piece of the first questionnaire.

An example of a piece of this first AHP questionnaire is shown in Figure 3:

These answers mean this particular expert agrees that in order to evaluate the touch of an orange

- firmness is very strongly more important than its skin roughness;
- firmness is moderately more important than defects in touch;
- defects in touch are moderately more important than skin roughness.

We have used the 1-9 Saaty’s fundamental scale [47].

Once the answers were gathered from all the experts, they were processed with the help of the Superdecisions© software v. 2.4.0 (Creative Foundations, Pittsburgh, PA, USA). The results of the weightings of all the variables were analyzed both individually and as a group. In order to obtain the group judgments, the geometric mean of the individual results was calculated. The results are presented in Table 1.

From the results of the criteria weights, we can conclude that the most important criteria for the expert tasters when evaluating the quality of an orange were the intensity of its fruity smell (17.58%), its juiciness (16.51%) and its sweetness (16.10%). We can also conclude those that were less important for the tasters were the roughness of the skin (1.02%) and the color of the pulp (1.37%). The first stage of the methodology ended with the completion of these weights. This was the generic stage; thus, the list of criteria and their weights can be used in any orange prioritization process, regardless of the type of oranges being evaluated. In the next section, we will describe the steps carried out to apply the methodology to a group of oranges with a specific tasting panel of consumers.

### 4.4. Prioritization of Oranges by a Consumer Tasting Panel

To this end, an orange tasting panel was arranged for four different types of oranges with the help of 23 consumers. All of them were people from the area of Valencia, with a great knowledge as consumers of Valencian oranges. The session took place in February 2020, so we were in the middle of the orange season. Each orange was coded with a different label without indicating its origin or supplier: M1, M2, M3, M4.

- M1. Orange Navel from supplier 1.
- M2. Orange from supplier 2.
- M3. Orange Navel Late from supplier 3.
- M4. Orange Navel Late from supplier 4.

Each consumer had to evaluate the four oranges by means of the AHP method. The 23 tasting stations were prepared with each one of them prepared with the four oranges. All the tasting stands were identical.

Again, the AHP methodology was explained by the facilitators of the tasting session, and after that the consumers proceeded to pairwise compare all the oranges according to each of the quality criteria. It took them around 40 min to listen to the explanation and answer all the questions stated.

**Figure 4:**
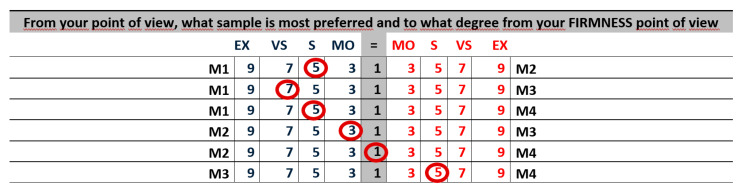
Example of a piece of the second questionnaire.

An example of a question of this particular questionnaire is shown in Figure 4.

These answers mean this particular consumer thought that, from the firmness perspective, their degree of preference among the four oranges was

- M1 is strongly more preferred than M2;
- M1 is very strongly more preferred than M3;
- M1 is strongly more preferred than M4;
- M2 and M4 are equally preferred;
- M4 is strongly more preferred than M3.

We have used the 1-9 Saaty’s fundamental scale [47].

**Figure 5:**
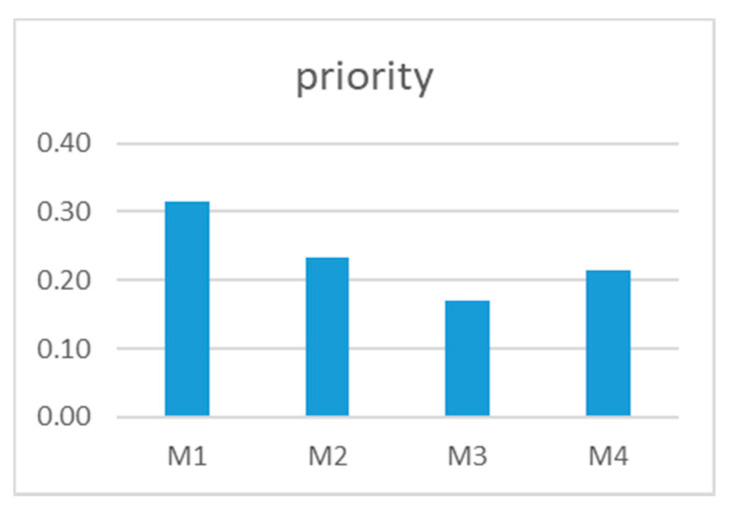
Results of the prioritization of the oranges by the consumers.

Once the answers were gathered from all the consumers, they were processed with the help of the Superdecisions© software v.2.4.0. The results of the prioritization of the oranges were analyzed both individually and as a group. The results are presented in Table 2 and Figure 5.

The results shown are the ones obtained by the whole group. For that, the individual judgments have been aggregated with the geometric mean. So, according to the orange consumers, the best orange was M1 (31%) followed by M2 (23%) and M4 (21%). The worst classified was M3 (17%). Since each of the oranges came from a different supplier, according to the consumers, the best orange supplier was supplier 1.

Once we reached this point, we further analyzed the performance of the participatory session in order to know how homogeneous the group was in its thinking. We investigated which were the pairwise comparison judgments that meant the greatest consensus and which were the ones that presented the greatest differences. This aspect is relevant if we want to further apply this methodology in other scenarios, with other types of oranges, or with other consumers. To achieve this, we used the geometric statistical descriptors following the concept of monogeneity defined by [52]. Monogeneity relates to the dispersion of the judgments around their geometric mean, i.e., how homogeneous the judgments of the members of a group are for each judgment they give in response to paired comparisons. This is done by deriving a measure of the dispersion of the judgments based on the geometric mean:

(1) $$\mu_{\mathrm{Gj}}=\pi_{i=1}^{n}x_{ij},$$

being *x_ij_* the judgement elicited by stakeholder *i* for pairwise comparison *j*. This measure we need is the geometric standard deviation:

(2) $$\sigma_{g}=\exp\sqrt{\frac{\sum_{i=1}^{n}{\left(\ln\frac{x_{ij}}{\mu_{Gj}}\right)}^{2}}{n}}$$

By analyzing the result of the geometric standard deviation, we can determine which of the judgements offered the lowest homogeneity and those that obtained the highest geometric standard deviation. The results of this analysis are presented in Table A1 (Appendix A). We present the geometric mean and standard deviation of each single pairwise comparison.

The following interesting results can be inferred from the analysis of Table A1. It seems that the less homogeneous cluster was acidity, followed by juiciness, because many of the group judgements offered a very low homogeneity among the different consumers. Instead, when moving to the presence of seeds cluster, we noticed a very high homogeneity. This is very easy to understand: oranges have or do not have seeds, and this is very easy to detect and, above all, to compare. That is, a high level of homogeneity indicates that the comparison judgement is easy to understand for the majority of the consumers, and thus answers are similar. On the other side, a low degree of homogeneity in eliciting the judgements implies that the question has been misunderstood or it is difficult to answer. Since all consumers compared a set of similar oranges, the judgements cannot differ very much.

A question difficult to answer poses a threat to the proper application of the AHP methodology. Such questions have to be further analyzed in order to understand the reasons.

## 5. Discussion

The aim of the paper is to create a consumer supplier selection model for fresh fruit, in particular Valencian oranges, for the retailer. With regard to RQ1, according to the expert panel, the most important criterion when evaluating the quality of an orange is the fruity smell (17.58%). The importance of this smell can be related to the freshness of the oranges. In other words, the more the orange smells, the fresher it looks. The second most important criterion is juiciness (16.51%). Regardless of taste, consumers could also relate the juiciness in the mouth to the freshness of the product. In third place comes sweetness with 16.10% of relative weight and, in fourth place, acidity with 12.37%. These two variables refer to the taste of the orange. It seems that tasters value sweetness more than acidity when it comes to consuming oranges. The fifth criterion is residue in the mouth (7.54%). The remaining criteria have a weight of less than 5%. The least important criteria for tasters are the roughness of the skin (1.02%) and the color of the pulp (1.37%). The most important criteria are related to the freshness and taste of the oranges according to [34]. These results are consistent with those obtained by [42] who found that consumers base the overall mandarin evaluation on juiciness, sweetness and acidity. In our case, aroma is more important. In reference to RQ2, we can see that there are differences among the clusters considered. Different criteria show different levels of homogeneity (answers to the pairwise comparison). The less homogeneous cluster is acidity followed by juiciness. In contrast, presence of seeds cluster is the most homogeneous. Therefore, we can conclude that acidity and juiciness are aspects difficult to assess.

The appearance of a fruit (related to its color and visual defects), as well as its organoleptic characteristics, flavor (sweetness, acidity, astringent, juiciness, etc.), texture (firmness, roughness and defects in touch) and smell are the main determinants of purchase intention [53,54] and, therefore, the consumer’s perception of a “quality” fruit. In this sense, sensory quality is a difficult term to define, as it relates both intrinsic product attributes and the interaction between the product and the consumer [37]. Thus, the use of sensory panels made up of more or less expert tasters is very common in the food industry, as they are capable of detecting product characteristics that would not be possible using instrumental techniques (relationship between objective parameters and consumer perception). Thus, some authors have related physicochemical parameters with consumer preferences (through sensory panels) in some fruits such as mandarins [55], apples [53,56,57], nectarines [58,59] and melons [37,60]. Therefore, if we want to include the consumer’s perception of the quality of a fruit in the supplier selection process, panels seem to be an appropriate methodology. However, as the consumer’s perception of a “quality” fruit depends on several variables, we need a methodology able to combine all of them and offer a final solution for the retailer to choose the right supplier. This was possible thanks to the AHP. When choosing among samples, the AHP method offers a prioritization of oranges based on the preferences of the consumer panel and the assessment of the criteria by the expert panel. Each of the samples was from a different supplier. This same prioritization can be used when choosing the supplier. In this way, we can choose the supplier that best suits the consumer’s preferences in terms of food quality. Our suggestion is to incorporate the combination of sensory panels and AHP into the selection process of fruit suppliers by retailers. Based on this approach, RQ3 can be answered positively.

From a managerial perspective, this selection process can help the agrifood sector in different ways up and down the supply chain [15,16,61,62]. First, farmers can better adapt varieties and harvesting times to consumer’s preferences. Second, storage times can also be adapted to the characteristics preferred by the consumer. Third, retailers can better understand what a consumer is looking for and develop a better selection of suppliers. However, the problems with the criteria obtained (juiciness, sweetness and acidity) are (i) they cannot be evaluated during the purchase process. This is a key question because consumers can modify their repurchase decision if they have a bad experience with the fruit. (ii) It is precisely these criteria where we find the greatest heterogeneity according to the results obtained. To solve this, retailers could design labels that offer information about these three attributes, for example with a scale from 1 (low) to 5 (high), at decision-making points. This would allow consumers to choose the citrus most suited to their preferences [30,42,55]. A further reflection should be carried out on the fact that a high heterogeneity has been found in these attributes. As the least homogeneous clusters are acidity followed by juiciness, it is necessary to further clarify the meaning of these attributes with consumers before starting the comparison procedure. This homogeneity aspect becomes very relevant since the methodology has the aim of being further generalized to broader contexts, for example, it could be implemented for the selection of other fruits and vegetables.

One of the limitations of the study is that we have only focused on the sensory quality of the fruit. There are other factors that determine the consumer’s purchase intention such as packaging [63,64,65], production type [34], price [66], food safety [67], consumer demographics [30,68] and personal situation [69]. In the case of exports, the image of the country of origin also influences the consumer’s decision [70]. As future lines of research, new multicriteria decision models could be developed that take into account all these variables.

## 6. Conclusions

In this paper, we have proposed a methodology for prioritizing oranges’ suppliers, incorporating the criteria used by the consumer to assess food quality (freshness, taste, and appearance), with the final aim of allowing retailers to choose their best supplier. The methodology has been based on the use of the organoleptic properties of the fruits and the application of the AHP multicriteria decision technique. This approach is absolutely necessary as consumers are willing to consume healthier products without compromising on taste.

We have divided the methodology into two stages. The first stage has been used to establish the decision model, that is, the decision criteria and their weights. This stage has been carried out with the collaboration of a group of 14 very experienced tasters. The model of criteria and their weights can therefore be considered a generic model that can be used in any future process of prioritization of oranges by consumers. The second stage is the prioritization of the oranges themselves. In our case study, we have worked with four different samples of oranges. They had different origins and were seasonal (February 2020). Each of the oranges was obtained from a different supplier.

The consumers, familiar with the consumption of oranges, were submitted to this new “tasting method” based on the AHP with the final objective of demonstrating that the methodology proposed could be carried out without complications, and that they found it useful and easy to develop.

Incorporating the methodology proposed in the supplier selection process by retailers can be very helpful as it can solve two big problems. First, consumption of fruits and vegetables can increase as retailers meet consumer demands. This is an important problem of public health, which is associated to obesity, cardiovascular diseases and unhealthy lifestyles if people do not consume the right proportion of fruits and vegetables. Second, as consumers find what they want in retail outlets, the waste of food can decrease, reducing its impact on the environment.

The correct performance of the proposed methodology has been validated by the expert tasters themselves and by consumers. In both cases, the people who collaborated in the study said that the methodology applied was simple, easy to understand and did not require much time. They all showed a high degree of satisfaction with the procedure. This indicates that the procedure could be replicable in future cases.

In future research, we propose to validate the prioritization of fruits obtained by consumers with the prioritization obtained by tasters. In this way, it could be concluded that the AHP orange prioritization methodology can replace the current expert orange tasting.

Finally, the application of the AHP methodology has helped to facilitate a participatory discussion among consumers on the concept of the quality of the oranges.

## Figures and Tables

**Table 1:** Weights of the orange quality criteria obtained by the group of tasting experts.

Criteria	Subcriteria	Weight (%)
C1. Touch	C11. Firmness	2.44
	C12. Skin roughness	1.02
	C13. Defects in touch	1.56
C2. Aspect	C21. Visual defects	4.25
	C22. Skin color	3.12
	C23. Pulp color	1.37
	C24. Easy peeling	2.65
	C25. Presence of seeds	3.11
	C26. Slices compaction	1.53
C3. Smell	C31. Herbaceous	2.50
	C32. Fruity	17.58
C4. Flavor	C41. Sweetness	16.10
	C42. Acidity	12.37
	C43. Bitter	3.08
	C44. Astringent	3.27
	C45. Residue in mouth	7.54
	C46. Juiciness	16.51
TOTAL		100.00

**Table 2:** Results of the prioritization of the oranges by the consumers.

Sample	Priority	Ranking
M1	0.31	1
M2	0.23	2
M3	0.17	4
M4	0.21	3

## Data Availability

Not applicable.

## Appendix A

In Table A1, we present the geometric mean and standard deviation of each single pairwise comparison.

**Table A1:** Geometric mean and geometric standard deviation of all the pairwise comparisons of the consumers.

Subcriteria	Pairwise Comparison	Geometric Mean	Geometric Deviation
C11. Firmness	M1-M2	1.31	3.96
	M1-M3	0.91	4.30
	M1-M4	0.79	3.75
	M2-M3	1.00	3.91
	M2-M4	0.59	3.91
	M3-M4	0.91	4.68
C12. Skin roughness	M1-M2	2.03	4.05
	M1-M3	1.34	4.34
	M1-M4	1.51	3.43
	M2-M3	0.71	3.96
	M2-M4	0.50	3.65
	M3-M4	1.00	4.29
C13. Defects in touch	M1-M2	1.98	3.69
	M1-M3	1.44	3.86
	M1-M4	1.54	3.16
	M2-M3	1.00	4.20
	M2-M4	0.73	3.98
	M3-M4	1.05	3.80
C21. Visual defects	M1-M2	2.40	2.97
	M1-M3	1.03	3.74
	M1-M4	1.10	4.61
	M2-M3	0.49	3.62
	M2-M4	0.48	3.93
	M3-M4	1.15	4.16
C22. Skin color	M1-M2	1.26	4.67
	M1-M3	1.84	3.44
	M1-M4	2.72	2.85
	M2-M3	1.68	3.36
	M2-M4	1.44	4.88
	M3-M4	1.92	3.30
C23. Pulp color	M1-M2	1.13	4.02
	M1-M3	1.81	3.66
	M1-M4	1.57	3.23
	M2-M3	1.72	3.66
	M2-M4	1.44	4.17
	M3-M4	0.48	2.78
C24. Easy peeling	M1-M2	0.96	3.98
	M1-M3	2.41	3.91
	M1-M4	2.43	3.39
	M2-M3	1.76	3.67
	M2-M4	2.00	3.64
	M3-M4	1.13	3.92
C25. Presence of seeds	M1-M2	1.09	2.01
	M1-M3	1.18	2.21
	M1-M4	0.91	2.00
	M2-M3	1.05	2.18
	M2-M4	0.94	1.77
	M3-M4	0.96	2.20
C26. Slices compaction	M1-M2	1.86	3.46
	M1-M3	2.63	3.15
	M1-M4	1.07	3.83
	M2-M3	1.29	3.81
	M2-M4	0.96	4.20
	M3-M4	0.62	3.19
C31. Herbaceous	M1-M2	1.93	3.22
	M1-M3	1.22	3.02
	M1-M4	1.49	3.07
	M2-M3	0.93	2.83
	M2-M4	0.67	2.76
	M3-M4	0.83	3.01
C32. Fruity	M1-M2	2.49	3.86
	M1-M3	2.30	2.62
	M1-M4	1.09	4.12
	M2-M3	1.19	3.25
	M2-M4	0.75	3.46
	M3-M4	0.57	3.83
C41. Sweetness	M1-M2	1.36	3.94
	M1-M3	2.07	3.76
	M1-M4	2.65	3.18
	M2-M3	1.68	4.06
	M2-M4	2.40	3.45
	M3-M4	1.16	4.56
C42. Acidity	M1-M2	1.11	4.02
	M1-M3	2.29	2.88
	M1-M4	1.29	4.07
	M2-M3	1.95	3.13
	M2-M4	1.16	4.01
	M3-M4	0.67	4.22
C43. Bitter	M1-M2	1.13	3.35
	M1-M3	1.69	2.60
	M1-M4	1.75	3.13
	M2-M3	1.24	2.54
	M2-M4	1.32	3.39
	M3-M4	0.94	3.51
C44. Astringent	M1-M2	1.09	2.80
	M1-M3	1.67	2.24
	M1-M4	1.55	2.47
	M2-M3	1.41	2.24
	M2-M4	1.12	2.87
	M3-M4	0.83	2.61
C45. Residue in mouth	M1-M2	1.43	2.91
	M1-M3	2.08	2.54
	M1-M4	1.54	3.17
	M2-M3	1.01	3.27
	M2-M4	0.94	3.28
	M3-M4	1.09	3.33
C46. Juiciness	M1-M2	1.11	4.50
	M1-M3	1.28	4.45
	M1-M4	1.14	3.74
	M2-M3	1.88	3.95
	M2-M4	1.11	3.95
	M3-M4	0.75	4.46

## References

[1] Steinmetz K.A., Potter J.D. (1991). Vegetables, fruit, and cancer. I. Epidemiology. Cancer Causes Control..

[2] van’t Veer P., Jansen M.C., Klerk M., Kok F.J. (2000). Fruits and vegetables in the prevention of cancer and cardiovascular disease. Public Health Nutr..

[3] World Health Organization The Global Strategy on Diet, Physical Activity and Health (DPAS). https://www.who.int/nmh/wha/59/dpas/en/.

[4] MAPA (2017). Estrategia Nacional de los Programas Operativos Sostenibles a Desarrollar por las Organizaciones de Productores de Frutas y Hortalizas.

[5] European Commission EUFV-CuTE | Chafea. Cultivating the Taste of Europe (CuTE). https://ec.europa.eu/chafea/agri/campaigns/eufv-cute.

[6] FAO (2020). Fruit and Vegetables—Your Dietary Essentials. The International Year of Fruits and Vegetables 2021. Background Paper.

[7] Capacci S., Mazzocchi M. (2011). Five-a-day, a price to pay: An evaluation of the UK program impact accounting for market forces. J. Health Econ..

[8] Erinosho T.O., Moser R.P., Oh A.Y., Nebeling L.C., Yaroch A.L. (2012). Awareness of the Fruits and Veggies-More Matters campaign, knowledge of the fruit and vegetable recommendation, and fruit and vegetable intake of adults in the 2007 Food Attitudes and Behaviors (FAB) Survey. Appetite.

[9] Rink S.M., Mendola P., Mumford S.L., Poudrier J.K., Browne R.W., Wactawski-Wende J., Perkins N.J., Schisterman E.F. (2013). Self-Report of fruit and vegetable intake that meets the 5 a day recommendation is associated with reduced levels of oxidative stress biomarkers and increased levels of antioxidant defense in premenopausal women. J. Acad. Nutr. Diet..

[10] MAPA (2020). Informe del Consumo de Alimentación en España 2019.

[11] MAPA (2020). Más Alimento, Menos Desperdicio.

[12] Saraiva A., Carrascosa C., Raheem D., Ramos F., Raposo A. (2020). Natural sweeteners: The relevance of food naturalness for consumers, food security aspects, sustainability and health impacts. Int. J. Environ. Res. Public Health.

[13] Aschemann-Witzel J., de Hooge I., Amani P., Bech-Larsen T., Oostindjer M. (2015). Consumer-Related food waste: Causes and potential for action. Sustainability.

[14] Raak N., Symmank C., Zahn S., Aschemann-Witzel J., Rohm H. (2017). Processing- and product-related causes for food waste and implications for the food supply chain. Waste Manag..

[15] Aschemann-Witzel J., Giménez A., Ares G. (2018). Consumer in-store choice of suboptimal food to avoid food waste: The role of food category, communication and perception of quality dimensions. Food Qual. Prefer..

[16] Parfitt J., Barthel M., MacNaughton S. (2010). Food waste within food supply chains: Quantification and potential for change to 2050. Phil. Trans. R. Soc. B.

[17] Berdegué J.A., Balsevich F., Flores L., Reardon T. (2005). Central American supermarkets’ private standards of quality and safety in procurement of fresh fruits and vegetables. Food Policy.

[18] Banaeian N., Mobli H., Nielsen I.E., Omid M. (2015). Criteria definition and approaches in green supplier selection—A case study for raw material and packaging of food industry. Prod. Manuf. Res..

[19] Lin P.C., Wu L.S. (2011). How supermarket chains in Taiwan select suppliers of fresh fruit and vegetables via direct purchasing. Serv. Ind. J..

[20] Liu A., Xiao Y., Ji X., Wang K., Tsai S.-B., Lu H., Cheng J., Lai X., Wang J. (2018). A novel two-stage integrated model for supplier selection of green fresh product. Sustainability.

[21] Rodas-González A., Huerta-Leidenz N., Jerez-Timaure N., Miller M.F. (2009). Establishing tenderness thresholds of Venezuelan beef steaks using consumer and trained sensory panels. Meat Sci..

[22] Saaty T.L. (1995). Decision Making for Leaders: The Analytic Hierarchy Process for Decisions in a Complex World.

[23] Saaty T.L. (2004). Decision making—the Analytic Hierarchy and Network Processes (AHP/ANP). J. Syst. Sci. Syst. Eng..

[24] Ortiz-Barrios M., Miranda-De la Hoz C., López-Meza P., Petrillo A., De Felice F. (2020). A case of food supply chain management with AHP, DEMATEL, and TOPSIS. J. Multi-Criteria Dec. Anal..

[25] Yadav S., Singh S.P. (2021). An integrated fuzzy-ANP and fuzzy-ISM approach using blockchain for sustainable supply chain. J. Enterp. Inf. Manag..

[26] Wang Y., Shi M., Liu H. Application of entropy-AHP-TOPSIS methods to select food suppliers. Proceedings of the 2017 International Conference on Humanities Science, Management and Education Technology (HSMET 2017).

[27] Haleem A., Khan S., Khan M.I. (2019). Traceability implementation in food supply chain: A grey-DEMATEL approach. Inf. Process. Agric..

[28] Govindan K., Kadziński M., Sivakumar R. (2017). Application of a novel PROMETHEE-based method for construction of a group compromise ranking to prioritization of green suppliers in food supply chain. Omega.

[29] Van Rijswijk W., Frewer L.J. (2008). Consumer perceptions of food quality and safety and their relation to traceability. Br. Food J..

[30] Baiardi D., Puglisi R., Scabrosetti S. (2016). Individual attitudes on food quality and safety: Empirical evidence on EU countries. Food Qual. Prefer..

[31] Zeithaml V.A. (1988). Consumer perceptions of price, quality, and value: A means-end model and synthesis of evidence. J. Mark..

[32] Grunert K.G., Larsen H.H., Madsen T.K., Baadsgaard A. (1996). Market. Orientation in Food and Agriculture.

[33] Nelson P. (1970). Information and Consumer Behavior. J. Polit. Econ..

[34] Petrescu D.C., Vermeir I., Petrescu-Mag R.M. (2020). Consumer understanding of food quality, healthiness, and environmental impact: A cross-national perspective. Int. J. Environ. Res. Public Health.

[35] Buitrago-Vera J., Escribá-Pérez C., Baviera-Puig A., Montero-Vicente L. (2016). Consumer segmentation based on food-related lifestyles and analysis of rabbit meat consumption. World Rabbit Sci..

[36] Puertas R., Marti L., Garcia-Alvarez-Coque J.-M. (2020). Food supply without risk: multicriteria analysis of institutional conditions of exporters. Int. J. Environ. Res. Public Health.

[37] Escribano S., Sánchez F.J., Lázaro A. (2010). Establishment of a sensory characterization protocol for melon (*Cucumis melo* L.) and its correlation with physical-chemical attributes: Indications for future genetic improvements. Eur. Food Res. Technol..

[38] Bettini M., Shaw P., Lanças F. (1998). Sensory and analytical evaluations of Brazilian orange juices and aromas. Qual. Control..

[39] Baxter I.A., Easton K., Schneebeli K., Whitfield F.B. (2005). High pressure processing of Australian navel orange juices: Sensory analysis and volatile flavor profiling. Innov. Food Sci. Emerg. Technol..

[40] Chen S., Nussinovitch A. (2001). Permeability and roughness determinations of wax-hydrocolloid coatings, and their limitations in determining citrus fruit overall quality. Food Hydrocoll..

[41] Pointer M.R., Attridge G.G. (1997). Some aspects of the visual scaling of large colour differences. Color. Res. Appl..

[42] Poole N.D., Martínez-Carrasco L., Vidal F. (2007). Quality perceptions under evolving information conditions: Implications for diet, health and consumer satisfaction. Food Policy.

[43] Lotong V., Chambers D.H., Dus C., IV E.C., Civille G.V. (2002). Matching results of two independent highly trained sensory panels using different descriptive analysis methods. J. Sens. Stud..

[44] Kim K.-O., O’Mahony M. (1998). A new approach to category scales of intensity I: Traditional versus rank-rating. J. Sens. Stud..

[45] Lotong V., Chambers E., Chambers D.H. (2003). Categorization of commercial orange juices based on flavor characteristics. J. Food Sci..

[46] Costell E. (2002). A comparison of sensory methods in quality control. Food Qual. Prefer..

[47] Saaty T.L. (1980). The Analytic Hierarchy Process.

[48] Pipatprapa A., Huang H.H., Huang C.H. (2018). Enhancing the effectiveness of AHP for environmental performance assessment of Thailand and Taiwan’s food industry. Environ. Monit. Assess..

[49] Song H., Lu B., Ye C., Li J., Zhu Z., Zheng L. (2021). Fraud vulnerability quantitative assessment of Wuchang rice industrial chain in China based on AHP-EWM and ANN methods. Food Res. Int..

[50] Ramos M.O., da Silva E.M., Lima-Júnior F.R. (2020). A fuzzy AHP approach to select suppliers in the Brazilian food supply chain. Production.

[51] García-Melón M., Gómez-Navarro T., Acuña-Dutra S. (2010). An ANP approach to assess the sustainability of Tourist strategies for the coastal national parks of Venezuela. Technol. Econ. Dev. Econ..

[52] Saaty T.L., Vargas L.G. (2007). Dispersion of group judgments. Math. Comput. Model..

[53] Iglesias I., Echeverría G., Soria Y. (2008). Differences in fruit colour development, anthocyanin content, fruit quality and consumer acceptability of eight “Gala” apple strains. Sci. Hortic..

[54] Moser R., Raffaelli R., Thilmany-Mcfadden D. (2011). Consumer Preferences for Fruit and Vegetables with Credence-Based Attributes: A Review. Int. Food Agribus. Manag. Rev..

[55] Campbell B.L., Nelson R.G., Ebel R.C., Dozier W.A., Adrian J.L., Hockema B.R. (2004). Fruit quality characteristics that affect consumer preferences for satsuma mandarins. HortScience.

[56] Harker F.R., Kupferman E.M., Marin A.B., Gunson F.A., Triggs C.M. (2008). Eating quality standards for apples based on consumer preferences. Postharvest Biol. Technol..

[57] Iglesias I., Echeverría G., Lopez M.L. (2012). Fruit color development, anthocyanin content, standard quality, volatile compound emissions and consumer acceptability of several “Fuji” apple strains. Sci. Hortic..

[58] Echeverría G., Cantín C.M., Ortiz A., López M.L., Lara I., Graell J. (2015). The impact of maturity, storage temperature and storage duration on sensory quality and consumer satisfaction of “Big Top^®^” nectarines. Sci. Hortic..

[59] Lopez G., Hossein Behboudian M., Echeverria G., Girona J., Marsal J. (2011). Instrumental and sensory evaluation of fruit quality for “Ryan’s sun” peach grown under deficit irrigation. Horttechnology.

[60] Lázaro A., De Lorenzo C. (2015). Texture analysis in melon landraces through instrumental and sensory methods. Int. J. Food Prop..

[61] Magalhães V.S.M., Ferreira L.M.D.F., Silva C. (2021). Using a methodological approach to model causes of food loss and waste in fruit and vegetable supply chains. J. Clean. Prod..

[62] Castle W.S., Baldwin J.C. (2011). Young-Tree performance of juvenile sweet orange scions on swingle citrumelo rootstock. HortScience.

[63] Karnal N., Machiels C.J.A., Orth U.R., Mai R. (2016). Healthy by design, but only when in focus: Communicating non-verbal health cues through symbolic meaning in packaging. Food Qual. Prefer..

[64] Machiels C.J.A., Karnal N. (2016). See how tasty it is? Effects of symbolic cues on product evaluation and taste. Food Qual. Prefer..

[65] van Rompay T.J.L., Deterink F., Fenko A. (2016). Healthy package, healthy product? Effects of packaging design as a function of purchase setting. Food Qual. Prefer..

[66] Marian L., Chrysochou P., Krystallis A., Thøgersen J. (2014). The role of price as a product attribute in the organic food context: An exploration based on actual purchase data. Food Qual. Prefer..

[67] Baselice A., Colantuoni F., Lass D.A., Nardone G., Stasi A. (2017). Trends in EU consumers’ attitude towards fresh-cut fruit and vegetables. Food Qual. Prefer..

[68] Wekeza S., Sibanda M. (2019). Factors influencing consumer purchase intentions of organically grown products in shelly centre, port shepstone, South Africa. Int. J. Environ. Res. Public Health.

[69] Carroll K.A., Samek A., Zepeda L. (2018). Food bundling as a health nudge: Investigating consumer fruit and vegetable selection using behavioral economics. Appetite.

[70] Knight J., Holdsworth D., Mather D. (2007). Determinants of trust in imported food products: Perceptions of European gatekeepers. Br. Food J..

